# Contribution of the Lipopolysaccharide to Resistance of *Shigella flexneri* 2a to Extreme Acidity

**DOI:** 10.1371/journal.pone.0025557

**Published:** 2011-10-03

**Authors:** Mara Martinić, Anilei Hoare, Inés Contreras, Sergio A. Álvarez

**Affiliations:** Departamento de Bioquímica y Biología Molecular, Facultad de Ciencias Químicas y Farmacéuticas, Universidad de Chile, Santiago, Chile; Monash University, Australia

## Abstract

*Shigella flexneri* is endemic in most underdeveloped countries, causing diarrheal disease and dysentery among young children. In order to reach its target site, the colon, *Shigella* must overcome the acid environment of the stomach. *Shigella* is able to persist in this stressful environment and, because of this ability it can initiate infection following the ingestion of very small inocula. Thus, acid resistance is considered an important virulence trait of this bacterium. It has been reported that moderate acid conditions regulate the expression of numerous components of the bacterial envelope. Because the lipopolysaccharide (LPS) is the major component of the bacterial surface, here we have addressed the role of LPS in acid resistance of *S. flexneri* 2a. Defined deletion mutants in genes encoding proteins involved in the synthesis, assembly and length regulation of the LPS O antigen were constructed and assayed for resistance to pH 2.5 after adaptation to pH 5.5. The results showed that a mutant lacking O antigen was significantly more sensitive to extreme acid conditions than the wild type. Not only the presence of polymerized O antigen, but also a particular polymer length (S-OAg) was required for acid resistance. Glucosylation of the O antigen also contributed to this property. In addition, a moderate acidic pH induced changes in the composition of the lipid A domain of LPS. The main modification was the addition of phosphoethanolamine to the 1′ phosphate of lipid A. This modification increased resistance of *S. flexneri* to extreme acid conditions, provide that O antigen was produced. Overall, the results of this work point out to an important role of LPS in resistance of *Shigella flexneri* to acid stress.

## Introduction

Species of the genus *Shigella* are the cause of shigellosis, a diarrheal disease that is endemic in developing countries [1]. The major site of *Shigella* pathology is the colon, where bacteria invade the intestinal mucosa, spread to the adjacent epithelial cells and cause much tissue damage, fluid secretion and inflammation, producing the clinical manifestations of shigellosis: diarrhea with blood and mucus [2]. Before colonization and invasion of the colonic mucosa, *Shigella* must overcome gastric acidity. In contrast to other bacterial enteric pathogens, such as *Vibrio cholerae* or *Salmonella* Typhi, species of *Shigella* are able to initiate infection of humans following ingestion of 10 to 100 bacteria, without neutralization of gastric acid [3]–[5]. For this reason, acid survival is considered an important pathogenic characteristic of these species [4].

*Shigella* has evolved sophisticated mechanisms for acid resistance that are induced under conditions of moderate acidity (pH 5.5) and are essential for its subsequent resistance to extreme acidity (pH 2.5). Two major acid-resistance pathways have been described in *Shigella*. The acid-resistance pathway 1, or oxidative system, is similar to that of *Escherichia coli* and requires complex media, oxidative growth and acid induction. This system is regulated by the RpoS sigma factor and the cyclic AMP receptor protein. The acid-resistance pathway 2, in turn, is a stationary-phase, glutamate-dependent pathway induced by growth in mildly acidic condition in the absence of oxygen [6]–[8]. A third system, which is induced in minimal media under fermentative conditions, has been described in *S. flexneri* 2457T [8].

Global gene expression profiling of the pH response in *S. flexneri* 2a [9], *E*. *coli* K-12 [10] and *Campylobacter jejunii* [11] has revealed a large number of genes that are differentially regulated by medium pH. The most pH-dependent genes were those involved in energy metabolism, as well as heat shock and oxidative stress genes. Also, pH differentially regulated a large number of periplasmic proteins and envelope components. This was not unexpected, since surface structures are exposed to the external pH and, thus, most probably play a role in the protective response of the cell to pH variations. Among *S. flexneri* pH-regulated genes, porin *ompF* and other outer membrane proteins genes were identified [9]. In *E. coli*, expression of transport proteins, redox modulators, chaperones and other membrane and periplasmic proteins was found to be pH-dependent [10], whereas in *C. jejunii*, capsular, flagellar and chemotaxis proteins expression was influenced by acid shock [11]. In *Helicobacter pylori*, acidity down-regulated 12 putative outer membrane proteins [12] and, in *Vibrio cholerae*, the OmpT porin was repressed by acid while the OmpU porin was required for acid resistance [13]. In addition, proteins involved in production of long fatty acids have been shown to be acid-induced in *Streptococcus mutans* [14], [15] and *Lactobacillus lactis* [16]. In the latter, proteomic analysis revealed that several proteins involved in the biosynthesis of the cell wall and of capsular polysaccharides were regulated by the pH of the medium [16].

The lipopolysaccharide (LPS) is the major component of the outer membrane that mediates bacterial interaction with the environment; however, not much is known about the role that this complex glycolipid plays in resistance to extreme acidity. LPS is composed of three covalently-linked domains: lipid A, which is embedded in the outer membrane, the central oligosaccharide core and the O polysaccharide or O antigen (OAg), which is exposed to the bacterial surface. In *S. flexneri*, the OAg has two preferred chain lengths, a short OAg (S-OAg) of 11 to 17 repeat units and a very long OAg (VL-OAg) of about 90 repeat units. These chain lengths, or modal distributions, are controlled by the WzzB and Wzz_pHS2_ regulators, respectively [17], [18]. Also, the addition of glucose residues to the OAg changes its conformation making it more compact and short [19].

It has been described that, under moderate acid conditions, the lipid A domain of LPS is modified by the addition of polar groups that vary between species. For instance, in *E. coli* and *Salmonella* Typhimurium, mildly acidic pH (6.0) activates the PmrAB two-component system. The PmrA transcription factor activates the expression of the *eptA* and *arnT* genes, which code for enzymes that attach phosphoethanolamine (PEtN) and L-amino arabinose (L-Ara4N), respectively, to the lipid A [20]–[25]. L-Ara4N is added to the 4′ phosphate of lipid A and sometimes to the 1′ phosphate, while PEtN can be attached to the 1 position. In *Salmonella*, but not in *E. coli*, PmrAB can be indirectly activated by low pH through the PhoPQ two-component system, which responds to low Mg^2+^ and Ca^2+^ and to low pH growth conditions [26], [27]. PhoPQ activation results in addition of palmitate to form hepta-acylated lipid A, incorporation of 2-OH myristate at position 3′ and removal of 3-OH myristate from position 3 of lipid A [25], [28]. In vivo, these changes in LPS structure are believed to contribute to bacterial resistance to antimicrobial peptides and to the moderate acidic environment found within macrophages [21], [25], [28], [29].

With regard to the OAg, a few studies have suggested that the structure of this domain of LPS is modified in response to the pH of the medium. Delgado et al [30] reported that PmrAB activation in *S.* Typhimurium results in upregulation of the *cld* (*wzz*_ST_) gene, increasing the L-OAg chain length distribution [30], whereas in *H. pylori*, exposure to low pH induced the expression of one unidentified gene related to OAg biosynthesis. A mutant lacking this gene was significantly more sensitive to acid stress (pH 3.5) than the wild-type strain. In addition, changes in the LPS electrophoretic profiles were observed [31]. The authors suggested that these modifications could contribute to the adaptation to resist extreme acidity, but this was not verified.

Together, these studies demonstrate that moderate low pH induce modifications of LPS in other enteropathogens, increasing their resistance to this environmental condition. However, they have not addressed whether these and/or other alterations of LPS structure play a role in resistance to extreme acid conditions. In this paper, we establish that both the OAg and the lipid A domains of *S. flexneri* LPS are modified by exposure to moderate acid conditions, we determine the nature of these modifications and show their importance in the acid-resistance phenotype of this pathogen.

## Results

### The O antigen contributes to acid resistance of *S. flexneri*

**Figure 1:**
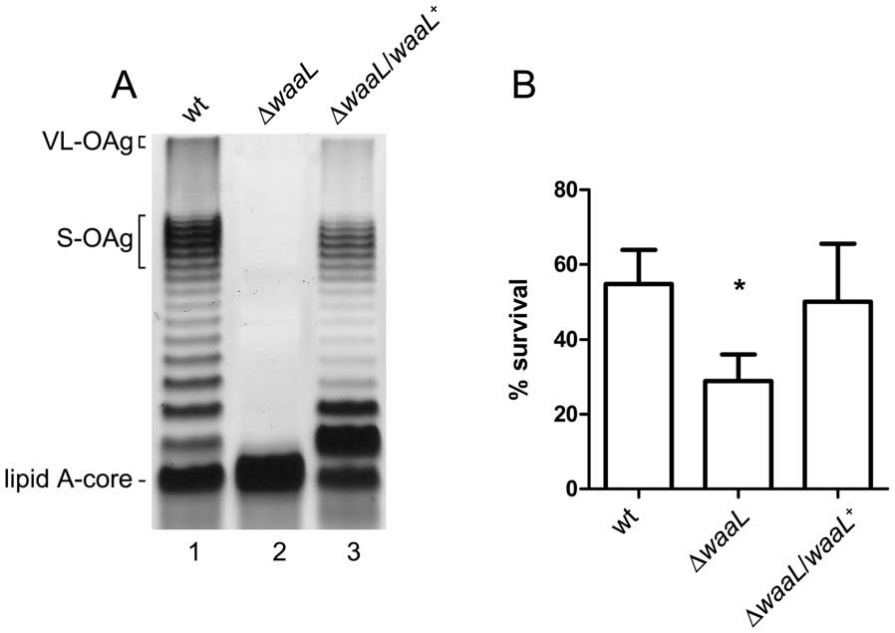
Contribution of the O antigen to *S. flexneri* 2a acid resistance. LPS profiles (A) and acid resistance (B) of S. *flexneri* 2457T (wt), MSF1210 (Δ*waaL*) and MSF1210/pMM112 (Δ*waaL*/*waaL* ^+^). LPS samples from equal numbers of bacterial cells (1×10^7^ CFU) were loaded in each lane and were analyzed by Tricine-SDS-polyacrylamide gel electrophoresis on a 14% (w/v) acrylamide gel followed by silver staining. Brackets indicate the VL-OAg, the S-OAg and the lipid A-core region. For acid resistance assays, cells were grown overnight in citrate-buffered LB (pH 5.5) and diluted 1∶1000 into the acid-challenge media. Survival is stated as a percentage of the inoculum. Averages±standard errors (error bars) are shown. Statistical significance was determined by a Student's *t* test. (*, *P* <0.05).

To ascertain whether the OAg is required for acid resistance, we constructed a deletion mutant of the *waaL* gene, which encodes the OAg ligase. The Δ*waaL* mutant (strain MSF1210) showed a rough LPS profile (devoid of OAg) in silver stained polyacrilamide gels (**Figure 1A**, lane 2). When this mutant was transformed with the intact *waaL* gene cloned in a multicopy plasmid (strain MSF1210/pMM112), an LPS profile showing the ladder corresponding to the OAg bands was observed (**Figure 1A**, lane 3). This profile was similar to that of the wild type (**Figure 1A**, lane 1). We compared the ability of the Δ*waaL* mutant and the wild-type 2457T to survive an exposure to pH 2.5 for 30 min, after overnight adaptation to pH 5.5, as described in Materials and Methods. As shown in **Figure 1B**, the mutant lacking OAg was significantly more sensitive to extreme acidity than the wild-type strain. The mutant harboring pMM112 recovered the ability to survive under this condition. A similar result was obtained for Δ*waaL* and Δ*waaL*/pMM112 derivatives of *S. flexneri* serotype 5a strain M90T (data not shown). Thus, the presence of polymerized OAg molecules increases resistance to extreme acidity of *S. flexneri* previously adapted to moderate acid pH.

### The S-OAg is required for acid resistance

**Figure 2:**
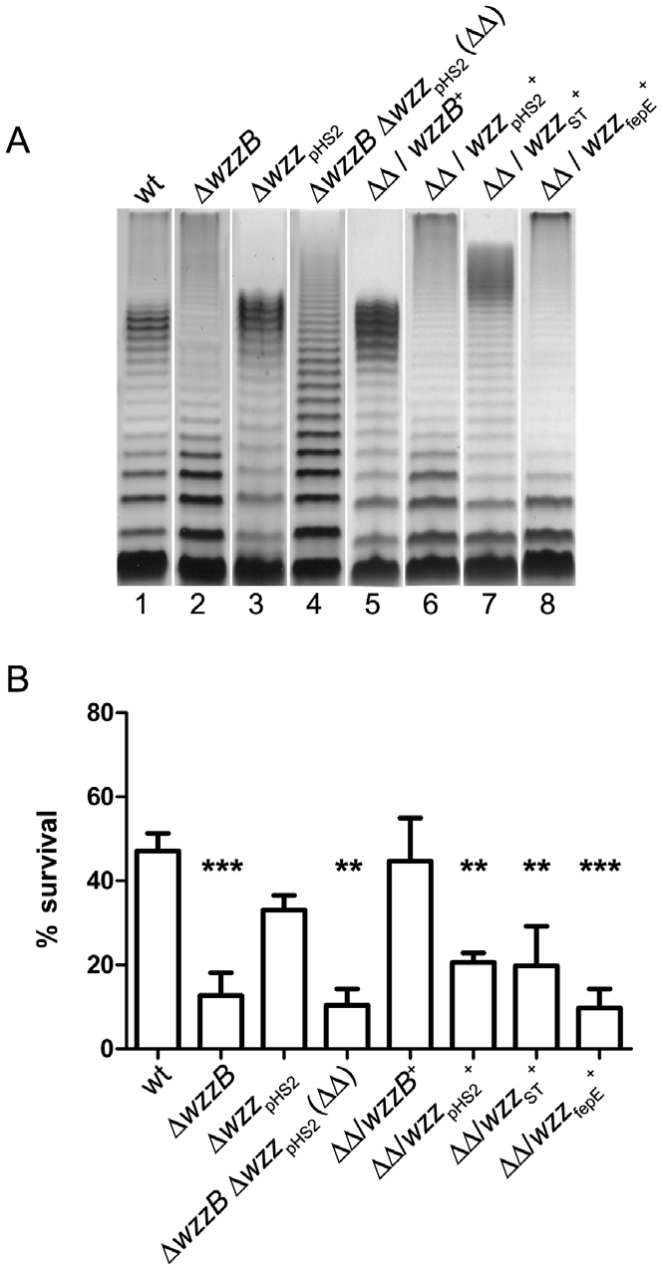
The *S. flexneri* S-OAg is required for acid resistance. LPS profiles (A) and acid resistance (B) of *S. flexneri* 2457T, mutants in the chain length regulators and mutants complemented with homologous or heterologous chain length regulators. Strains are 2457T (wt), MSF102 (Δ*wzzB*), MSF107 (Δ*wzz*_pHS2_::*aph*), MSF209 (Δ*wzzB* Δ*wzz*_pHS2_::*aph*), MSF209/pJC139, MSF209/pJC144, MSF209/pMM110 and MSF209/pJC142. LPS samples from equal numbers of bacterial cells (1×10^7^ CFU) were loaded in each lane and were analyzed by Tricine-SDS-polyacrylamide gel electrophoresis on a 14% (w/v) acrylamide gel followed by silver staining. For acid resistance assays, cells were grown overnight in citrate-buffered LB (pH 5.5) and diluted 1∶1000 into the acid-challenge media. Survival is stated as a percentage of the inoculum. Averages±standard errors (error bars) are shown. Statistical significance was determined by a Student's *t* test. (**, *P* <0.01, ***, *P* <0.001).

Because the results described above indicated that the OAg contributes to acid resistance, we investigated whether a particular length of the OAg molecules was required for this property. For this purpose, we constructed defined deletion mutants in genes *wzzB* (strain MSF102) and *wzz*_pHS2_ (strain MSF107), which encode proteins that regulate the S-OAg and VL-OAg chain length distributions, respectively. We also generated a double mutant lacking both regulators (strain MSF209). LPS was obtained and analyzed by SDS-PAGE and silver staining. As shown in **Figure 2A**, the Δ*wzzB* mutant was devoid of S-OAg but it produced VL-OAg (lane 2), whereas the Δ*wzz*_pHS2_ mutant exhibited the opposite phenotype (lane 3). The double mutant showed a random distribution of OAg molecules (lane 4), in contrast to the wild type that produced both S-OAg and VL-OAg (lane 1). Transformation of the double mutant with each intact gene cloned in a multicopy plasmid restored the expression of the corresponding chain length (lanes 5 and 6). Acid resistance of each of these strains was assayed for 30 min at pH 2.5 after overnight adaptation to pH 5.5. As shown in **Figure 2B**, Δ*wzzB* and the double mutant (strains MSF102 and MSF209, respectively) were significantly more sensitive to extreme acid conditions, with survival levels less than 20% of the wild type. In contrast, the Δ*wzz*_pHS2_ mutant (strain MSF107) showed acid resistance levels similar to the wild type. In accordance with these results, the double mutant transformed with a multicopy plasmid including the intact *wzzB* gene, but not the intact *wzz*_pHS2_ gene, recovered the wild-type levels of resistance to extreme acidity.

In order to ascertain whether the S-OAg chain length that is characteristic of *S. flexneri* 2a is required for acid resistance, strain MSF209 was transformed with multicopy plasmids expressing each OAg chain length regulator of *Salmonella* Typhimurium. Similar to *S. flexneri*, this bacterium shows a bi-modal distribution of the OAg expressing a long (L-)OAg of 16 to 35 repeat units regulated by Wzz_ST_ and a very long (VL-)OAg of more than 100 repeat units regulated by Wzz_fepE_ [32]–[36]. The *S. flexneri* double mutant expressing the heterologous chain regulators (strains MSF209/pMM110 and MSF209/pJC142) produced OAg modal lengths characteristic of *S*. Typhimurium (**Figure 2A**, lanes 7 and 8, respectively). The double mutant expressing any of the heterologous regulators showed acid resistance values significantly lower than those of the wild-type 2457T and of the mutant expressing *S. flexneri* WzzB (**Figure 2B**). Together, these results indicate that S-OAg distinctive of *S. flexneri* 2a is essential to confer acid resistance.

### Glucosylation of the O antigen also contributes to acid resistance

**Figure 3:**
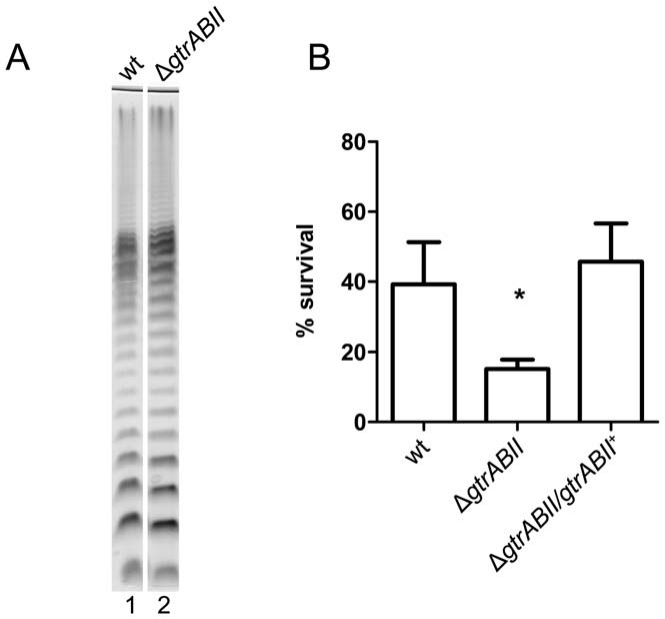
Glucosylation of the O antigen contributes to acid resistance. (A) LPS profiles of S. *flexneri* 2457T (wt) and MSF2743 (Δ*gtrABII*). LPS samples from equal numbers of bacterial cells (1×10^7^ CFU) were loaded in each lane and were analyzed by Tricine-SDS-polyacrylamide gel electrophoresis on a 14% (w/v) acrylamide gel followed by silver staining. (B) Acid resistance of S. *flexneri* 2457T (wt), MSF2743 (Δ*gtrABII*) and MSF2743/pMM111 (Δ*gtrABII*/*gtrABII* ^+^). Cells were grown overnight in citrate-buffered LB (pH 5.5) and diluted 1∶1000 into the acid-challenge media. Survival is stated as a percentage of the inoculum. Averages±standard errors (error bars) are shown. Statistical significance was determined by a Student's *t* test. (*, *P* <0.05).

The OAg of *S. flexneri* 2a is glucosylated in the last rhamnose residue [37]. It has been described that this modification affects the conformation of the polymer in such a way that LPS molecules containing glucosylated OAg are more compact and shorter than molecules of non-glucosylated OAg [38]. Because we found that the length of the OAg was critical to acid resistance (S-OAg), we decided to investigate whether the degree of glucosylation could also influence this property. To this end, we constructed a deletion mutant in the *gtrABII* operon encoding proteins responsible for glucosylation of the OAg. The electrophoretic profile of this mutant (strain MSF2743) showed OAg bands migrating slightly faster than those of the wild-type 2457T. This observation was more evident when the electrophoresis was performed on a longer polyacrylamide gel (**Figure 3A** **,** lanes 2 and 1, respectively). Acid resistance experiments showed that MSF2743 was about 50% more sensitive to pH 2.5 than the wild type. When this mutant was transformed with the intact *gtrABII* operon cloned in a multicopy plasmid, the acid resistant phenotype was restored to wild-type levels (**Figure 3B**).

### A mild acidic condition modifies the LPS structure of *S. flexneri*

**Figure 4:**
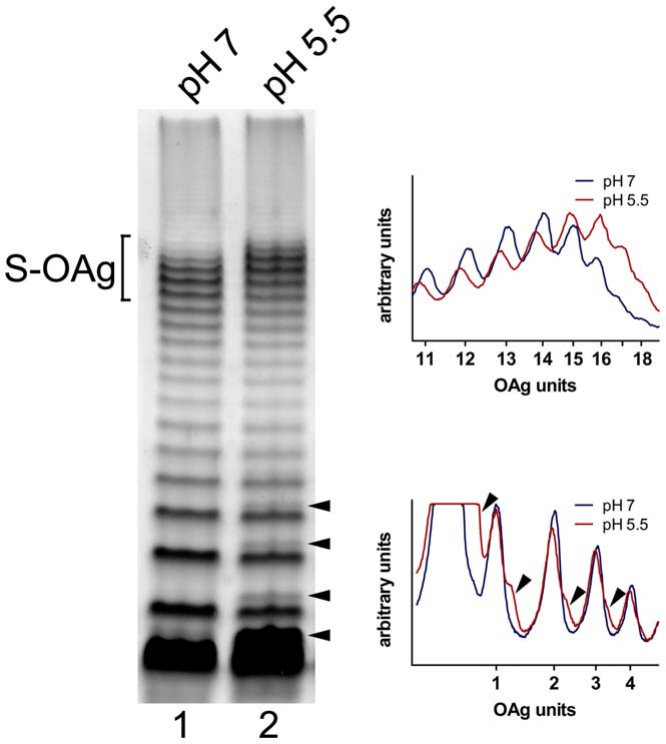
Effect of pH on LPS profiles from S. *flexneri* 2457T. Cells were grown at pH 7.0 or 5.5 and LPS samples were obtained. LPS samples from equal numbers of bacterial cells (1×10^7^ CFU) were loaded in each lane and were analyzed by Tricine-SDS-polyacrylamide gel electrophoresis on a 14% (w/v) acrylamide gel followed by silver staining. Bracket shows the S-OAg region. Arrow heads point to the double bands observed in LPS from bacteria grown at pH 5.5. The right panels show the densitograms of the bands in the gel. Upper graph shows the bands corresponding to the S-OAg (11 to 18 units) and lower graph shows the bands corresponding to the Lipid A-core substituted with 1 to 4 units.

**Figure 5:**
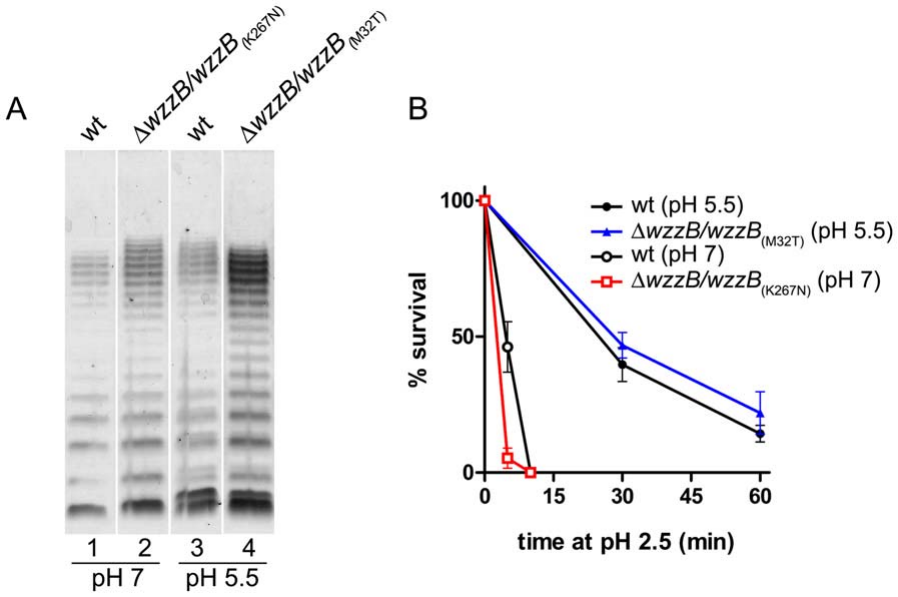
Changes in the electrophoretic mobility of the LPS induced by moderate acidic conditions are not relevant to resistance to extreme acid. LPS profiles (A) and acid resistance (B) of *S. flexneri* 2457T (wt) and MSF102 (Δ*wzzB*) transformed with plasmids pRMCD108 (WzzB_(K267N)_) or pRMCD127 (WzzB_(M32T)_). Bacteria were grown at pH 7.0 or 5.5 and LPS samples were obtained. LPS samples from equal numbers of bacterial cells (1×10^7^ CFU) were loaded in each lane and were analyzed by Tricine-SDS-polyacrylamide gel electrophoresis on a 14% (w/v) acrylamide gel followed by silver staining. For acid resistance assays, cells were grown overnight in citrate-buffered LB (pH 5.5) and diluted 1∶1000 into the acid-challenge media. Survival is stated as a percentage of counts at time zero. Averages±standard errors (error bars) are shown. Statistical significance was determined by the two-way ANOVA and Bonferroni post test. No significant differences were found in bacteria previously grown at pH 5.5 (closed symbols) or pH 7.0 (open symbols).

The mechanisms for resistance to extreme acidity are induced by previous adaptation to a moderate acidic pH [6]–[8]. In other enterobacteria, it has been demonstrated that this latter condition causes changes in the LPS structure [31]. We decided to investigate whether adaptation to pH 5.5 altered the LPS of *S. flexneri* 2a, and, if that was the case, whether these changes contributed to resistance to extreme acidity. We first compared the LPS profiles of bacteria grown at pH 7.0 and pH 5.5. As shown in **Figure 4**, the LPS of bacteria grown at pH 5.5 had a slightly lower mobility of the bands corresponding to S-OAg than bacteria grown at pH 7.0 (lanes 2 and 1, respectively). It was observed that at pH 5.5, this region contained lower amounts of OAg bands ranging from 11–15 units, but higher amounts of OAg bands of 16–18 units (see densitogram in **Figure 4**, upper right). In order to ascertain whether this modification was relevant to acid resistance, we performed acid-resistance assays using the Δ*wzzB* mutant (strain MSF102) transformed with plasmids containing site-directed mutations in the gene encoding the WzzB regulator (**Table 1**). When strain MSF102/pRMCD108 (mutation WzzB_(K267N)_) was grown at pH 7.0, produced an LPS profile similar to that observed in the wild type grown at pH 5.5 (**Figure 5A**, compare lanes 2 and 3). In contrast, when strain MSF102/pRMCD127 (mutation WzzB_(M32T)_) was grown at pH 5.5, it produced an LPS profile similar to the wild type grown at pH 7.0 (**Figure 5A**, compare lanes 1 and 4). Thus it was expected that, if the shift of the S-OAg that occurs at pH 5.5 was relevant to acid resistance, strain MSF102/pRMCD108 would be resistant to extreme acid pH without previous adaptation to pH 5.5. On the other hand, strain MSF102/pRMCD127 would be sensitive to acid even after adaptation.

**Table 1:** Evaluation of bacterial cell surface charge.

Strain (growth pH)	% Cytochrome C bound
2457T (7.0)	27.1±2.5
2457T (5.5)	8.8±2.4
2457T/pMM113 (7.0)	15.0±3.8

Values represent percent binding of cytochrome C after incubation with bacteria for 10 min at room temperature. Data are the means and standard deviations from two independent experiments run in duplicate.

The results showed that MSF102/pRMCD108 was as sensitive as the wild-type strain grown at pH 7.0 (non-adapted), whereas MSF102/pRMCD127 showed resistance levels similar to the wild-type grown at pH 5.5 (adapted) (**Figure 5B**). These results indicated that the change in the electrophoretic mobility of the S-OAg induced by moderate acidic conditions is not relevant to resistance to extremely acidic pH.

**Figure 6:**
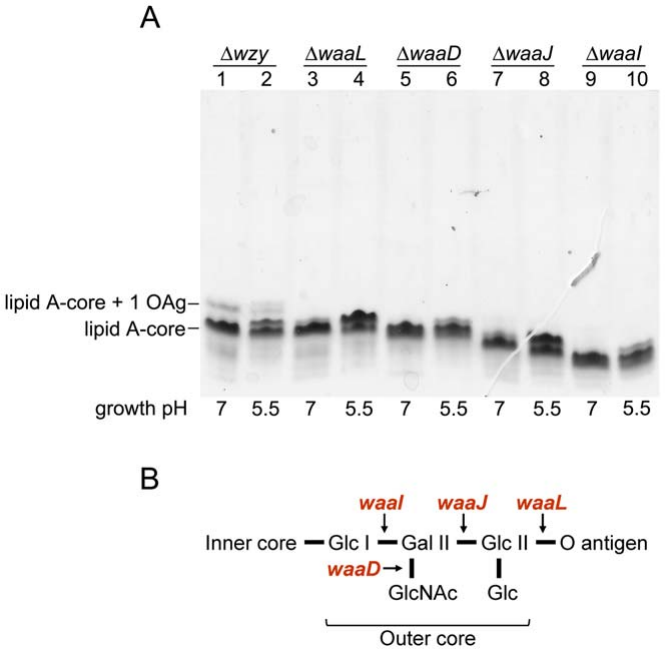
LPS profiles from S. *flexneri* mutants defective in O antigen and core synthesis and proposed structure of the outer core of *S. flexneri* 2a. (A) Bacteria were grown at pH 7.0 or 5.5. LPS samples from equal numbers of bacterial cells (1×10^7^ CFU) were loaded in each lane and were analyzed by Tricine-SDS-polyacrylamide gel electrophoresis on a 14% (w/v) acrylamide gel followed by silver staining. The strains are MSF1749 (Δ*wzy*), MSF1210 (Δ*waaL*), MSF1144 (Δ*waaD*), MSF1009 (Δ*waaI*) and MSF1015 (Δ*waaJ*). (B) Genes encoding proteins involved in the addition of the different sugars during outer core biosynthesis are indicated in red.

We also noticed that the OAg of bacteria grown at acid pH migrated as double bands (**Figure 4**, see densitogram lower right). The double bands were not attributable to glucosylated OAg molecules because they were also present in the Δ*gtrABII* mutant grown at pH 5.5 (data not shown). The double bands were more evident in the OAg molecules containing one, two, three and four repeat units and in the lipid A-core region. The double band in this region was also evident in LPS obtained form bacteria grown at pH 7.0, although it was much fainter than at pH 5.5 (compare lanes 1 and 2 in **Figure 4** **,** and lanes 1 and 3 in **Figure 5**). With these observations, we hypothesized that this modification actually occurred in the lipid A-core region and, as a consequence, the OAg profile appeared altered. In order to test this notion, we analyzed the LPS profiles of defined mutants in the polymerization or ligation of the OAg, and with different degrees of truncation of the outer core (Δ*waaD*, Δ*waaJ* and Δ*waaI*). The LPS patterns of the mutants and the structure of the LPS outer core [39], [40] are shown in **Figures 6A and B**, respectively. The Δ*wzy* mutant exhibited the lipid A-core region substituted by one OAg unit (lanes 1 and 2), whereas the Δ*waaL* mutant synthesized only lipid A-core (lanes 3 and 4). The Δ*waaD* mutant showed a core region that migrated slightly faster than that of Δ*waaL* (lanes 5 and 6) consistent with the lack of the GlcNAc residue in the outer core (**Figure 6B**). Deletion of *waaJ* and *waaI* genes resulted in additional truncations of the core region (lanes 7 and 8; 9 and 10, respectively). The *waaJ* gene product adds Glc II while the *waaI* product adds the Gal residue to the outer core (**Figure 6B**). All strains exhibited the double band when grown at pH 5.5. These results indicated that the changes in the LPS structure by exposure to moderate acidic condition affected the lipid A and/or the inner core regions of LPS.

In *S*. Typhimurium, moderate acidic pH activates the PhoPQ two-component system. Phosphorylated PhoP activates expression of the *pagP* gene, encoding a protein responsible for the addition of palmitate to lipid A [25], [41], [42]. To define whether a similar regulation occurs in *S. flexneri*, we constructed a *phoP* deletion mutant and analyzed the LPS profiles of bacteria grown at pH 7.0 and 5.5. The results showed that double bands were still present in the Δ*phoP* mutant (data not shown). Thus, it was unlikely that the double bands were due to the addition of palmitate to lipid A.

**Figure 7:**
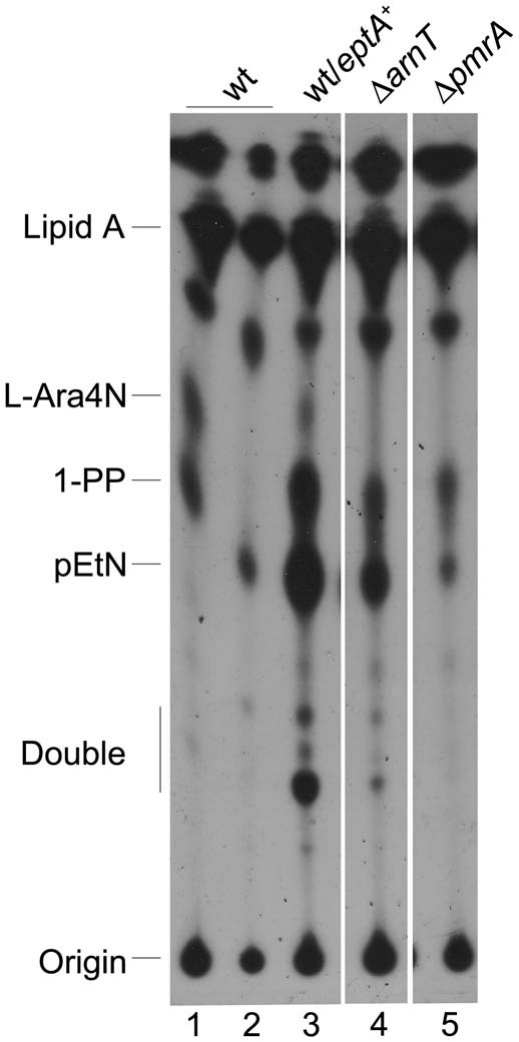
Effect of pH on lipid A modifications in *S. flexneri*. ^32^P-labelled lipid A was isolated from bacteria grown in N-minimal medium at pH 7.0 (lanes 1, 3, 4 and 5) or 5.5 (lane 2). Lipid A species were resolved by TLC with the solvent system chloroform/pyridine/88% formic acid/water (50∶50∶16∶5, v/v). The strains are 2457T (wt), 2457T/pMM113 (wt/*eptA* ^+^), MSF1650 (Δ*arnT*) and MSF1666 (Δ*pmrA*).

To elucidate the chemical nature of the modifications of lipid A, *S. flexneri* was grown in LB broth in the presence of ^32^Pi, at pH 7.0 and pH 5.5. The ^32^P-labelled lipid A was isolated and subjected to TLC as described in Materials and Methods. The results showed that *S. flexneri* grown at pH 7.0 produces lipid A molecules partially substituted with a second phosphate group (1-PP) and L-Ara4N (**Figure 7**, lane 1). In contrast, when bacteria were grown at pH 5.5 the lipid A was modified by the addition of PEtN, while 1-PP and L-Ara4N were not detected (**Figure 7**, lane 2). The chemical nature of these species was confirmed by analyzing the lipid A profiles of the Δ*arnT* mutant (strain MSF1650) and of the wild-type strain transformed with the *eptA* gene cloned in a multicopy plasmid (strain 2457T/pMM113) grown at pH 7.0. This strategy was used because we were unable to obtain an *eptA* mutant either by the Red swap methodology or by using the suicide vector pGP704 containing an internal region of the gene.

In strain 2457T/pMM113, spots corresponding to lipid A substituted with one and two molecules of PEtN (double) were observed. As in the wild type grown at pH 7.0, 1-PP and L-Ara4N were also detected (**Figure 7**, lane 3). This last spot was missing in the chromatogram of lipid A from strain MSF1650 (**Figure 7**, lane 4). Interestingly, in this mutant genetic background, PEtN was detected in lipid A from bacteria grown at pH 7.0. Finally, we analyzed lipid A obtained from a Δ*pmrA* mutant (strain MSF1666). As expected, this mutant lacked L-Ara4N; however, it was able to add PEtN to lipid A at pH 7.0 (**Figure 7**, lane 5).

### A less negative bacterial surface charge is produced by lipid A modification under mild acidic condition

To evaluate whether the modifications of lipid A by the addition of polar groups alter the cell surface charge, we measured the cytochrome C binding affinity of the wild type cells grown at pH 7.0 and 5.5. The results (**Table 1**) showed that binding was lower when bacteria were grown at pH 5.5 that at pH 7.0, suggesting that addition of PEtN to lipid A at moderate acidic pH confers a less negative charge to the bacterial surface. To confirm this notion, cytochrome C binding affinity of the wild type cells overexpressing the *eptA* gene, grown at pH 7.0, was measured. As shown in **Table 1**, these cells bound about one half the amount of cytochrome C than the wild type grown under this condition.

### Lipid A modifications induced under mild acidic conditions confer resistance to extreme acidity

**Figure 8:**
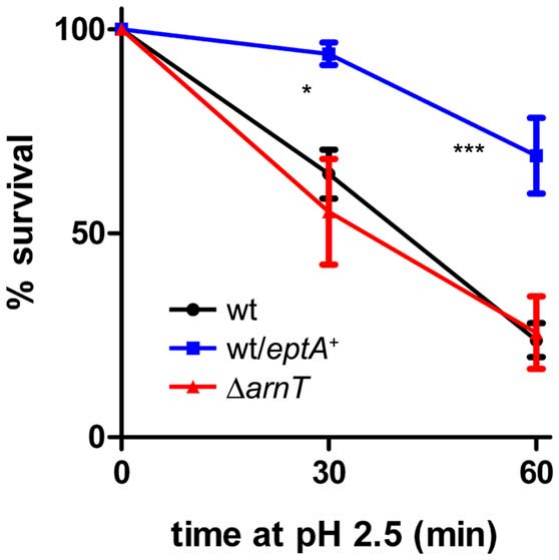
Effect of lipid A modifications on acid resistance of *S. flexneri.* The strain are 2457T (wt), 2457T/pMM113 (wt/*eptA* ^+^) and MSF1650 (Δ*arnT*). Cells were grown overnight in citrate-buffered LB (pH 5.5) and diluted 1∶1000 into the acid-challenge media. Survival is stated as a percentage of counts at time zero. Averages±standard errors (error bars) are shown. Statistical significance was determined by. the two-way ANOVA and Bonferroni post test (*, *P* <0.05, ***, *P* <0.001).

To investigate whether the modification of the lipid A region induced by growth at pH 5.5 is involved in resistance to extreme acid conditions, we tested acid resistance of the wild-type strain transformed with pMM113. As shown in **Figure 8**, overexpression of the *eptA* gene significantly increased acid resistance after 30 and 60 min of exposure to pH 2.5, compared to the parental strain. This result indicates that the addition of PEtN to the lipid A region of LPS, induced by a moderate pH, contributes to *S. flexneri* resistance to extreme acid. In contrast, deletion of the *arnT* gene did not alter this property. As shown in **Figure 8**, the Δ*arnT* mutant (strain MSF1650) showed levels of acid resistance identical to the wild-type strain.

Finally, we transformed the Δ*waaL*, Δ*wzzB* and Δ*grtABII* mutants with plasmids pMM112 or pMM113 and assayed the acid resistance of the resulting strains. The results showed that the transformed strains were as sensitive to extreme acidity as the original mutants (data not shown), indicating that, even though lipid A modification contributes to acid resistance, the presence of glucosylated OAg is fundamental to this property.

## Discussion

Remodelling of the bacterial envelope is an important strategy used by bacteria to adapt to stressful conditions. Global expression studies carried out in other enteropathogens have revealed that a great number of genes involved in the biosynthesis of surface structures are regulated by the pH of the environment [9]–[13]. In the present study, we investigated whether the lipopolysaccharide of *S. flexneri* 2a is modified by exposure to a moderate acidic pH, and if these modifications increase bacterial resistance to extreme acidity.

Our results clearly show that the presence of OAg is required for acid resistance, since a mutant that does not bind the polymer to the lipid A-core was highly sensitive to pH 2.5, even after being adapted to pH 5.5. These results are consistent with the work of other authors, who demonstrated that the lack of OAg or diminished amount of this polysaccharide impaired the ability of *Rhizobium leguminosarum* biovar trifolii [43], *H. pylori* [31] and *Salmonella enterica* serovar Dublin [44] to grow or persist under acidic conditions. In an *E. coli* O157:H7, a strain closely related to *Shigella*, it has also been demonstrated the involvement of LPS in the resistance to organic acid [45]. Although the acid stress used in that study was different from our conditions (pH 4.5 versus pH 2.5), their results support our conclusion that the OAg is essential to acid resistance. More interestingly, we found that a particular length of the OAg, the S-OAg modal distribution, is required for resistance to extreme acidity. In contrast, neither the VL-OAg modal length nor OAg lengths characteristic of *S*. Typhimurium contributed significantly to this property.

McGowan et al [31] proposed that the OAg could act as a physical barrier to prevent entry of protons to the bacterial cell. Our results do not agree with this notion, because the mutant lacking VL-OAg (Δ*wzz*_pHS2_) was as resistant to extreme acid as the wild-type strain, meaning that LPS molecules of high molecular weight do not confer higher levels of resistance, as would be expected if the OAg polymer acted as a physical barrier for protons.

Another explanation for the role of the OAg in the acid stress response could be that the polysaccharide may be positively charged repelling protons from the surrounding medium. However, at the pH values used in this study, the –OH groups of the rhamnose residues in the *S. flexneri* OAg are protonated and neutral [46] and, hence, they do not confer a positive charge to the OAg molecules. On the other hand, the amide group in the N-acetylglucosamine residue is deprotonated and neutral at the pH values used here. In consequence, the OAg would not be charged under the conditions of this study and, thus, it would not act as an electrostatic barrier for the influx of protons.

Because it is known that moderate acidic conditions produce changes in the expression of outer membrane proteins [47], [48] and that the absence of some porins affects resistance to acidic pH [49], [50], we propose that the presence of OAg, in particular the S-OAg modal length, may be necessary for assembly or function of a protein in the bacterial envelope required for acid resistance. In support of this idea, it has been suggested that a preferential association between certain outer membrane proteins and LPS molecules of a particular length may occur [35]. For example, the IcsA protein, involved in actin polymerization that allows *S. flexneri* spreading between epithelial cells, requires the presence of S-OAg to be assembled at one pole of the bacterial surface [35, 51 52]. Alternatively, or in addition, synthesis of S-OAg may slow down polymerization of very long OAg molecules (VL-OAg), which could mask a membrane protein needed for acid resistance. In this context, our previous work [53] demonstrated that the two *Shigella* chain-length regulators compete to control the degree of O antigen polymerization; hence, increase in one modal length results in a parallel decrease in the other.

Our results also show that glucosylation of the OAg contribute to acid resistance. Our data indicates that this modification is also present in bacteria grown under standard conditions (LB, pH 7.0), in agreement with a previous study conducted by West et al [19]. Interestingly, these authors demonstrated that glucosylation of the OAg is required for the proper function of the Type Three Secretion System (T3SS) of *S. flexneri*, because glucosylated OAg is more compact and short than non-glucosylated LPS, allowing a proper exposure of the needle complex [38]. Additionally, a more compact and dense OAg favours the interaction between OAg molecules, contributing to stabilize the outer membrane [54] and protecting it from the damage caused by acidic pH and entry of protons. Given all these observations, we speculate that glucosylated OAg containing mainly S-OAg molecules is required for optimal functioning of one or more proteins that contribute to the acid stress response.

The current work also demonstrates that moderate acidic pH induces modifications in the lipid A region of *S. flexneri*. Previous work by Gibbons et al [29] showed that lipid A of *S*. Typhimurium is covalently modified by the addition of acyl and/or polar substituents in response to low Mg^2+^ concentrations and mild acidic pH. Under these conditions, lipid A was derivatized with phosphoethanoamine (PEtN), aminoarabinose (L-Ara4N), 2-hydroxymyristate and/or palmitate moieties. It is believed that these modifications confer resistance to cationic antimicrobial peptides by masking negative phosphate groups with positively charged moieties [55]–[58]. In *E. coli* K12 W3110, lipid A is modified in response to growth under acidic but no low Mg^2+^ conditions. The main change is the addition of PEtN to the phosphate molecule at position 1 of N-acetyl glucosamine. The addition of two PEtN to each phosphate molecule at positions 1 and 4 was also detected in a small fraction of lipid A molecules. In contrast to *S*. Typhimurium, no L-Ara4N was detected. At pH 7.5, lipid A was mainly unmodified or, a low fraction of molecules, carried a 1-PP group [29], [59].

Our results demonstrate that similar modifications occur in *S. flexneri*. As in *E. coli*, the main change induced at pH 5.5 was the addition of PEtN to lipid A, whereas L-Ara4N was not detected. In contrast, at pH 7.0 the lipid A molecules were derivatized with 1-PP and L-Ara4N. The regulatory pathways involved in these modifications also appear to be different to *E. coli* and *S*. Typhimurium, because a Δ*pmrA* mutant lacked L-Ara4N but it was able to add PEtN to lipid A, showing a chromatographic profile similar to Δ*arnT*. This result suggests that modification of lipid A with PEtN in response to acid pH is not mediated by the PmrAB system. Future work will be needed to uncover the regulatory system involved in this modification.

The presence of L-Ara4N does not seem to add to extreme acid resistance. In contrast, addition of PEtN to lipid A protects *S. flexneri* from extreme acid pH. It has been reported that PEtN lipid A modification reduces the net charge of the phosphate group from −1.5 to −1 at neutral pH [54]. Here we demonstrate that addition of PEtN confers a less negative charge to bacterial surface. We speculate that at pH 2.5 the presence of positively charged amine groups increases the net charge to reach positive values. Thus, we hypothesize that lipid A modification with polar substituents generate an electrostatic barrier that acts repelling protons and diminishing their influx to the bacterial cytoplasm.

To our knowledge, this is the first report that addresses the role of LPS in resistance of *S. flexneri* to extreme acid conditions. Our data demonstrate that both modification of the lipid A region and the presence of glucosylated OAg molecules containing the S-OAg modal length are required to confer *S. flexneri* its high level of acid resistance. Our results strengthen the hypothesis proposed by others [35], [38] that, during evolution of *S. flexneri* as a successful pathogen, selection of preferred OAg chain lengths and glucosylation of the polysaccharide was optimized. LPS modifications that occur in response to environmental conditions contribute to adaptation of bacteria to the different stages of infection.

## Materials and Methods

### Bacterial strains, plasmids, media and growth conditions

**Table 2** summarizes the properties of the bacterial strains and plasmids used in this study. Bacteria were grown at pH 7.0 in Luria-Bertani medium (LB, 10 g/l Bacto tryptone, 5 g/l Bacto yeast extract, 5 g/l NaCl) or in citrate-buffered LB medium (pH 5.5). Ampicillin (100 µg/ml), kanamycin (50 µg/ml), and chloramphenicol (20 µg/ml) were added when appropriate.

**Table 2:** Strains and plasmids used in this study.

Strain or plasmid	Relevant properties^a^	Source or reference
***S. flexneri***		
2457T	wild-type strain	ISP^b^
MSF107	2457T Δ*wzz*_pHS2_::*aph*, Km^R^	[50]
MSF102	2457T Δ*wzzB*	[50]
MSF209	2457T Δ*wzzB* Δ*wzz*_pHS2_::*aph*, Km^R^	[50]
MSF1749	2457T Δ*wzy*	This study
MSF1210	2457T Δ*waaL*	This study
MSF1144	2457T Δ*waaD*	This study
MSF1009	2457T Δ*waaI*	This study
MSF1015	2457T Δ*waaJ*	This study
MSF2743	2457T Δ*gtrABII*	This study
MSF1650	2457T Δ*arnT*	This study
MSF1666	2457T Δ*pmrA*	This study
**Plasmids**		
pKD46	*bla* PBAD *gam bet exo* pSC101 *ori*TS	[57]
pKD4	*bla* FRT *aph* FRT PS1 PS2 *ori*R6K	[57]
pCP20	*bla cat c*I857 λP_R_ *flp* pSC101 *ori*TS	[58]
pGEM-T-Easy	TA cloning vector, Ap^R^	Promega
pJC139	*S. flexneri* 2457T *wzzB* cloned into pGEM-T-Easy Ap^R^	[63]
pJC142	*S*. Typhimurium LT2 *wzz*_fepE_ cloned into pGEM-T-Easy Ap^R^	[67]
pMM110	*S*. Typhimurium LT2*wzz*_LT2_ cloned into pGEM-T-Easy, Ap^R^	This study
pJC144	*S*. *flexneri* 2457T *wzz*_pHS2_ cloned into pGEM-T-Easy, Ap^R^	[66]
pMM112	*S*. *flexneri* 2457T *waaL* cloned into pGEM-T-Easy, Ap^R^	This study
pMM113	*S. flexneri* 2457T *eptA* cloned into pGEM-T-Easy, Ap^R^	This study
pCC1	Single copy cloning vector, oriII, oriV, Cm^R^	EPICENTRE
pMM111	*S. flexneri* 2457T *gtrABII* cloned into pCC1, Cm^R^	This study
pRMCD108	WzzB_(K267N)_	[68]
pRMCD127	WzzB_(M32T)_	[68]

a Km^R^, kanamycin resistant; Ap^R^ ampicillin resistance; Cm^R^, chloramphenicol resistance.

b ISP, Institute of Public Health, Santiago, Chile.

### Mutagenesis of waaD, waaJ, waaI, waaL, gtrABII, arnT and pmrA genes

Mutagenesis was performed by the Red/Swap method to create chromosomal mutations by homologous recombination using PCR products [60]. *Shigella flexneri* 2a strain 2457T containing pKD46, which expresses the λ Red recombinase system, was transformed with PCR products that were generated using as template plasmid pKD4, which contains the FRT-flanked kanamycin-resistance gene (*aph*). Primers were designed according to the DNA sequence available for the *S. flexneri* 2457T strain. Each primer pair also carried 30 bases that were homologous to the edge of the gene targeted for disruption. The sequences of the oligonucleotide primers are shown in **Table 3**. The kanamycin-resistant transformants were replica plated in the absence of antibiotic selection at 37°C and finally assayed for ampicillin sensitivity to confirm loss of pKD46. To obtain non-polar deletion mutants, the antibiotic resistance gene was removed by transforming the gene replacement mutants with pCP20, which encodes the FLP recombinase [61]. Correct insertional gene replacements and the deletion of the antibiotic gene cassettes were confirmed by PCR.

**Table 3:** Primers used in this study.

primers	Sequence (5′-3′)	purpose
W_sf_waaL1	acctcaacattatttttctctctcgagaaacatatgaatatcctccttag	*waaL* deletion
W_sf_waaL2	cttgtttttcatcgctaataataagccggcgtgcaggctggagctgcttc	*waaL* deletion
Sf_waaL-1	atttaacggcggcactggat	*waaL* cloning into pGEM-T Easy
Sf_waaL-2	tcactacagttgggatggcg	*waaL* cloning into pGEM-T Easy
Fwr WgtrABII	tgtaaagtacacatctataggtgtgctgaagtgcaggctggagctgcttc	*gtrABII* deletion
Rev WgtrABII	Ccttttttctctgatagttttattatcacccatatgaatatcctccttag	*gtrABII* deletion
SfgtrABII- HindIII (1)	cccaagcttggggcgagcaaaatcgatgcgat	*gtrABII* cloning into pCC1
SfgtrABII- HindIII (2)	cccaagcttgggtgaaaaagggaggcgctcttt	*gtrABII* cloning into pCC1
1149	ggctacactgtctccagcttcatcc	*wzz*ST cloning into pGEM-T Easy
1150	gagcaccatccggcaaagaagcttac	*wzz*ST cloning into pGEM-T Easy
eptA (1)	ccggggcgaaagaggttatg	*eptA* cloning into pGEM-T Easy
eptA (2)	cgccagaatcagtccctgca	*eptA* cloning into pGEM-T Easy
Wzysf_A	tcaataacttccctatttttaacatcctttattttgctcccatatgaatatcctccttag	*wzy* deletion
Wwzysf_B	Ctgattattggtggtggtggaagattactggagccatgtgtaggct	*wzy* deletion
W_sf_waaD1	gttgataaaataatatttacggttactcctcatatgaatatcctccttag	*waaD* deletion
W_sf_waaD2	ttcaaaccaattatgaataacctcttcgaagtgtaggctggagctgcttcg	*waaD* deletion
W_sf_waaJ1	gattttaaacatcttactcaatttaaagatcatatgaatatcctccttag	*waaJ* deletion
W_sf_waaJ2	acctttcataacattatatttaattgctgtgtgtaggctggagctgcttcg	*waaJ* deletion
W_sf_waaI1	tctcaactcaatgatagtgacatcatccttcatatgaatatcctccttag	*waaI* deletion
W_sf_waaI2	gaagcatttttctttataatactttaaatagtgtaggctggagctgcttcg	*waaI* deletion
arnT (H1+P1)	gtagtcgctgatgaaatcggtacgttaccttatcggcatcgtgcaggctggagctgcttc	*arnT* deletion
arnT (H2+P2)	gttagccagatcatttgggacgatactgaatcagcaccagcatatgaatatcctccttag	*arnT* deletion
WpmrA (1)	gagtgagtaaatgaaaattctgattgttgaagacgatacggtgcaggctggagctgcttc	*pmrA* deletion
WpmrA (2)	gattcaattagttttcctcattcgcgaccagcatatagcccatatgaatatcctccttag	*pmrA* deletion

Underline indicates the region that anneals to the 5′ or 3′ end of the antibiotic resistance cassette used for the mutagenesis.

### Construction of expression plasmids

DNA fragments containing the *S. flexneri* 2457T *waaL*, *arnT*, *eptA*, *gtrABII* genes and *S*. Typhimurium LT2 *wzz*_ST_ were obtained by PCR. The amplicons were cloned into pGEM-T Easy as recommended by the supplier, except for *gtrABII* operon that was cloned into the one copy vector pCC1 (**Table 1**).

### LPS analysis

Culture samples were adjusted to an optical density at 600 nm of 2.0 in a final volume of 100 µl. Then, proteinase K-digested whole-cell lysates were prepared as described previously [62], and LPS was separated on 12% (w/v) acrylamide gels using a Tricine-sodium dodecyl sulfate (SDS) buffer system [63]. Gel loadings were normalized so that each sample represented the same number of cells. Gels were silver stained by a modification of the procedure of Tsai & Frasch [64]. Densitometry analysis was performed using the UN-SCANT-IT gel software (Silk Scientific).

### Acid resistance assay

For acid-resistance assays bacteria were aerobically grown overnight in LB (pH 7.0) or citrate-buffered LB (pH 5.5) media. Cultures grown to stationary phase were diluted 1∶1000 into 5 ml of E medium (0.2 g/l MgSO4·7H_2_O, 2 g/l citric acid monohydrate, 10 g/l K_2_HPO_4_·3 H_2_O_,_ 3.5 g/l NaNH_4_HPO_4_·4H_2_O), acidified with HCl to pH 2.5. This medium was supplemented with 0.2% glucose as carbon source, 20 µg/ml aspartic acid and 20 µg/ml nicotinic acid. The pH 2.5 cultures were incubated at 37°C with shaking; acid challenge was stopped at 0 and 30 min by dilution in LB and cell viability was immediately determined by plating appropriate dilutions in LB plates in triplicate. At least three repetitions were performed for each experiment. Acid resistance was calculated as percent survival, taking the bacterial counts obtained at time 0 as 100%.

### Analysis of ^32^Pi labelled lipid A

An overnight culture grown at 37°C on LB was diluted 1000-fold into 5 ml of fresh LB medium at pH 7.0 or 5.5. Labelling was started by addition of 5 µCi/ml of ^32^H_3_PO_4_ (Chilean Commission of Nuclear Energy), and allowed to continue growing overnight at 37°C with shaking. The ^32^P-labeled bacteria were collected by centrifugation and washed twice with 5 ml of phosphate-buffered saline (PBS), pH 7.4. The bacterial pellets were resuspended in 0.8 ml of PBS and the lipid A fractions were extracted as described by Zhou et al [65]. The lipid A samples were dissolved in chloroform/methanol (4∶1, v/v), and several microliters (5,000 cpm per lane) were applied to the origin of a Silica Gel 60 TLC plate. The ^32^P-labelled lipid A species were resolved with the solvent system chloroform/pyridine/88% formic acid/water (50∶50∶16∶5, v/v). The plate was developed by autoradiography.

### Cytochrome C binding assay

An overnight culture grown at 37°C on LB was diluted 1000-fold into 5 ml of fresh LB medium at pH 7.0 or 5.5. Bacteria were collected by centrifugation and washed twice with 10 mM phosphate buffer (PB), pH 6.8. Bacterial pellets were resuspended in 1 ml of PB and the OD_600 nm_ was measured. Then, samples were diluted to 5 OD units and 30 µl of cytochrome C (10 mg/ml in PB) were added to 1.2 ml of bacterial suspensions. Samples were incubated for 10 min at room temperature and then centrifuged for 2 min at 13,000 rpm. The supernatants (1 ml) were recovered and the OD_530 nm_ was measured.

## Aknowledgments
